# Enhancing Smoking Cessation Support: Audit of Nicotine Replacement Therapy (NRT) in Psychiatric Inpatient Care

**DOI:** 10.1192/bjo.2025.10591

**Published:** 2025-06-20

**Authors:** Nikhil Gauri Shankar, Hadiya Kar, Wamiqur Rehman, Aanika Hoque, Ishraq Elahi, Charles Okwuchi, Rajkumar Reddy

**Affiliations:** 1 Betsi Cadwaladr University Health Board, Wrexham, United Kingdom; 2 Betsi Cadwaladr University Health Board, Bangor, United Kingdom

## Abstract

**Aims:** This audit aimed to evaluate the prescription practices of Nicotine Replacement Therapy (NRT) in adult psychiatry inpatient wards in North Wales. Given the high prevalence of smoking among individuals with mental health disorders, the Welsh Government’s legislation mandating smoke-free hospital grounds underscores the importance of effective NRT use to support smoking cessation and improve mental and physical health outcomes. Specifically, the audit assessed adherence to key standards, including timely provision of NRT, assessment of smoking status, and documentation of contraindications and drug interactions.

**Methods:** The audit reviewed all patients admitted to the inpatient psychiatry wards over one week, with no exclusion criteria. Data were collected from patients’ paper records and drug charts using a standardized audit proforma. The standards evaluated included: (1) documentation of smoking status, (2) provision of NRT within four hours of admission, (3) documentation of contraindications, and (4) consideration of drug interactions. Compliance percentages were calculated to identify areas for improvement.

**Results:** The audit revealed that smoking status was assessed in 79% of patients, while NRT was offered within the four-hour target in 59% of cases. However, contraindications and drug interactions were considered in just 14% of cases. While NRT was generally offered to most patients, significant gaps were identified in documenting contraindications and drug interactions.

**Conclusion:** This audit highlights the effective use of NRT in more than three-quarters of cases but underscores a need for improved compliance in documenting contraindications and drug interactions when prescribing NRT in psychiatric inpatient settings. Recommendations include enhancing clinician awareness and training on best practices for NRT documentation and developing a standardized tool to streamline and ensure adherence to these standards. Continued evaluation and refinement of NRT practices in psychiatric care can contribute to better health outcomes for patients with mental health disorders, aligning with public health efforts to create smoke-free healthcare environments.

